# Polygenic Epidemiology

**DOI:** 10.1002/gepi.21966

**Published:** 2016-04-07

**Authors:** Frank Dudbridge

**Affiliations:** ^1^ Department of Non‐communicable Disease EpidemiologyLondon School of Hygiene and Tropical MedicineKeppel StreetLondonUnited Kingdom

**Keywords:** missing heritability, genetic correlation, genetic risk prediction, Mendelian randomization

## Abstract

Much of the genetic basis of complex traits is present on current genotyping products, but the individual variants that affect the traits have largely not been identified. Several traditional problems in genetic epidemiology have recently been addressed by assuming a polygenic basis for disease and treating it as a single entity. Here I briefly review some of these applications, which collectively may be termed polygenic epidemiology. Methodologies in this area include polygenic scoring, linear mixed models, and linkage disequilibrium scoring. They have been used to establish a polygenic effect, estimate genetic correlation between traits, estimate how many variants affect a trait, stratify cases into subphenotypes, predict individual disease risks, and infer causal effects using Mendelian randomization. Polygenic epidemiology will continue to yield useful applications even while much of the specific variation underlying complex traits remains undiscovered.

The completion of genome‐wide association studies (GWAS) for hundreds of complex diseases and traits has created new challenges for genetic epidemiologists as we seek to understand the function of associated loci, using multiple emerging technologies [Ziegler and König, 2014]. At the same time, the realization that most if not all complex traits are polygenic—that is, they are influenced by thousands of genetic variants each having a small effect—has spurred the development of methods that address traditional problems in genetic epidemiology by treating the entire polygenic basis as a single entity. By dealing with the genetic basis *en masse*, these methods access more of the heritable component of complex traits than is possible by single‐variant approaches, and thus alleviate much of the missing heritability problem that has recently so exercised genetic epidemiologists [Maher, 2008]. Here I briefly review several such applications, which together form an emerging field of *polygenic epidemiology*.

The concept of a polygenic risk is well established in classical genetics and in humans has been applied for some time in, for example, segregation analysis of complex diseases [Antoniou et al., 2002; Risch, 1990]. But only with the development of genome‐wide panels of SNP markers has it become possible to treat the polygenic basis explicitly. An early success was in the recalculation of twin‐based heritabilities using observed, rather than expected, genetic similarity between dizygous pairs [Visscher et al., 2008]. Two influential papers then introduced complementary methods for demonstrating evidence of a polygenic effect, and have each led to further developments across a range of applications.

The polygenic scoring method was applied by Purcell et al. [2009] to argue that schizophrenia has a polygenic risk. Although their GWAS identified few individually significant SNPs, a small systematic increase in χ^2^ statistics was observed across the genome, and could not be reduced by control for population structure or genotyping error. The polygenic scoring method uses a GWAS dataset, called the *training sample*, to estimate effect sizes for each SNP, and to select SNPs according to a *P*‐value threshold. In a second dataset, called the *target sample*, a polygenic risk score is calculated for each subject as the weighted sum of risk alleles at the selected SNPs, with the weights being the effects estimated in the training data. A polygenic risk would cause this score to be associated for very liberal selection *P*‐values. Purcell et al. observed significant association of the score with a selection threshold as high as *P* < 0.5, and used simulation to argue that this was consistent with a polygenic effect; their argument has since been strengthened by theory [Dudbridge, 2013; Yang et al., 2011b].

Linear mixed models were used by Yang et al. [2010] to show that much of the heritability of height can be explained by current GWAS chips, even though very little could be explained by known associated variants. The approach derives from methods of quantitative genetics used in crop and livestock breeding, in which the genetic value (or breeding value) of an individual can be derived as a random effect by relating the phenotypes of the study subjects to their known pedigree structure. The innovation in human studies is that nominally unrelated subjects can be used, by estimating their (distant) relatedness from genome‐wide marker data. By relating genetic to phenotypic similarity across all pairs of subjects, the variance of the genetic values can be estimated and taken as an estimate of narrow‐sense heritability. Because this depends upon the markers used to estimate relatedness, especially the fact that they are usually selected to be common (e.g., >1% minor allele frequency), this estimate is known as *chip heritability*. A further advantage of using unrelated individuals is that shared environmental effects are minimal and therefore unlikely to bias the chip heritability.

Both polygenic scores and linear mixed models have been used to infer a polygenic basis for a wide range of traits [Bush et al., 2010; Lu et al., 2014; Speliotes et al., 2010; Visscher et al., 2012], to the degree that it is now generally accepted that all complex traits are polygenic. Much attention is now directed at demonstrating and estimating the genetic correlation between pairs of traits. This can be done with polygenic scores, by training the score on one trait and testing it against another: if there is no shared genetic basis, the score will not be associated. A bivariate linear mixed model may also be used, modeling the genetic variance for two traits simultaneously with their covariance. Both approaches have shown a shared basis for schizophrenia and bipolar disorder, as well as for other pairs of psychiatric disorders [Cross‐Disorder Group of the Psychiatric Genomics Consortium, 2013; Lee et al., 2013; Purcell et al., 2009]. This indicates a common molecular etiology for these conditions, which may prove useful for developing novel treatments or identifying individuals at risk, but may also suggest problems with current nosology and diagnosis. A shared basis has also been demonstrated for mammographic breast density and breast cancer, which may in part explain the former as a risk factor for the latter, by mediating some of the genetic risk of disease [Varghese et al., 2012]. On the other hand, genetic correlation has not been observed for some pairs of traits for which it might be expected, especially in neurology [Goris et al., 2014]. Autism appears to occupy a distinct position among psychiatric disorders in that it has no genetic correlation with the other major disorders studied, other than a low (16%) correlation with schizophrenia [Lee et al., 2013].

Linear mixed models give immediate estimates of chip heritability and genetic correlation: inference of the polygenic effect follows by testing these effects against their standard errors [Visscher et al., 2014]. By contrast, polygenic scoring gives a hypothesis test for the polygenic effect, without an immediate estimate of chip heritability. However, methods have been developed to infer chip heritability and genetic correlation from the result of a polygenic score test [Palla and Dudbridge, 2015; Stahl et al., 2012], by estimating under what degree of chip heritability would the observed result be expected. These models also allow for a proportion of variants with no effect on the trait; linear mixed models have also been implemented with this feature [Meuwissen et al., 2001; Moser et al., 2015; Zhou and Stephens, 2012]. A notable finding has been that the proportion of variants affecting a complex trait rarely exceeds 5%, even in heterogeneous samples [Palla and Dudbridge, 2015]. Thus the classical infinitesimal model may not hold in truth, although the traits remain highly polygenic. This finding affects the interpretation of genetic correlation, because the overall genetic correlation is determined by the proportion of variants with effects on both traits and the correlation of effects among those variants. A given genetic correlation may be concentrated among a few variants with highly concordant effects on both traits, or dispersed among more variants with only weakly concordant effects. Although the genetic correlation is informative at the whole subject level, it is not in itself very informative at the single‐variant level. Chip heritability itself is only interpretable in the context of an assumed model, usually (for disease traits) a liability threshold model, and the population‐specific environment [Hopper and Mack, 2015]; however it is generally useful to estimate the broad degree of genetic variance and correlation, even if it is challenging to provide or interpret a precise estimate.

A third approach to assessing chip heritability is linkage disequilibrium (LD) scoring [Bulik‐Sullivan et al., 2015a]. Its rationale is that the more variants a given marker is in LD with, the higher is its (marginal) association statistic likely to be. Regression of χ^2^ statistics for genome‐wide markers on their LD “scores” gives an estimate of chip heritability (the slope of the regression) as well as of systematic bias due to population structure (the intercept). An extension allows the estimation of genetic covariance between traits [Bulik‐Sullivan et al., 2015b].

A further application is the estimation of chip heritability within sub‐groups of markers. Such “genome partitioning” analyses have demonstrated that polygenic effects are spread uniformly across the chromosomes, but variants with known functional effects tend to explain more variation than others [Visscher et al., 2007; Yang et al., 2011a].

Polygenic scores show promise for patient stratification and subphenotyping. Hamshere et al. [2011] showed that, among bipolar disorder cases, polygenic scores for schizophrenia risk could distinguish schizo‐affective cases from others, while not distinguishing psychotic cases from nonpsychotic. In inflammatory bowel disease, polygenic scores can distinguish cases with colonic from ileal Crohn's disease, and from those with ulcerative colitis [Cleynen et al., 2016]. Prostate cancer screening targeted to men with high polygenic risk could reduce the rate of overdiagnosis [Pashayan et al., 2015].

Ultimately we might hope for individual risk prediction from polygenic analysis, which indeed first motivated the polygenic scoring method [Wray et al., 2007]. The performance of genetic prediction is bounded by the heritability [Clayton, 2009], but early attempts fell well short of that limit [Evans et al., 2009]. Furthermore, family history is an informative marker for genetic risk, and any predictor based on measured genotypes should exceed that benchmark to be deemed worthwhile [Aulchenko et al., 2009]. It is now clear that much larger samples are required to attain accurate prediction from polygenic models, of the order of 10^5^ subjects [Chatterjee et al., 2013; Daetwyler et al., 2008; Dudbridge, 2013]. The reason is simply that as more variants enter the prediction model (as is necessary to explain the genetic risk), the greater is the sampling error in the total score, so the latter must be kept extremely small at the single‐variant level. National biobanks and disease consortia are now beginning to approach this scale [CardiogramPlusC4D Consortium, 2015; Locke et al., 2015; Michailidou et al., 2013; Schizophrenia Working Group of the Psychiatric Genomics Consortium, 2014], and the outlook for genetic prediction is becoming more promising than has recently appeared.

A common practice is to perform genetic prediction using only variants that are robustly associated at genome‐wide significance [Mavaddat et al., 2015; Szulkin et al., 2015; Talmud et al., 2015]. This is based on intuition that the risk score contains genuine predictors and no “noise,” and could therefore be easily conveyed to clinicians and policy makers. However, precisely in line with the missing heritability problem, such predictors explain very little variation in disease risk, and therefore have little predictive accuracy. Analytic results [Dudbridge, 2013] suggest that, even at very large sample sizes, prediction is optimized by selecting variants with *P‐*values as high as 0.001 into a risk score (Fig. 1). Perhaps counterintuitively, it is possible to predict with accuracy close to the theoretical maximum even while including many neutral variants in the model. By the same token, as sample sizes continue to grow it should be possible to achieve useful levels of prediction even while many individually associated variants remain to be discovered. In translating such predictors into practice, it will be important to present the polygenic risk as a single entity in order to allay concerns that it contains many irrelevant variants. This is naturally achieved by linear mixed models, which estimate a single genetic value for each individual as a random effect, and these models are now being extended to allow for subgroups of variants with no or little effect [Moser et al., 2015; Speed and Balding, 2014].

**Figure 1:**
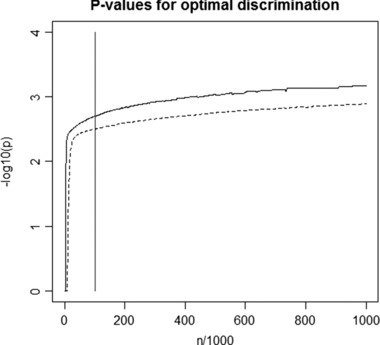
*P*‐values (−log_10_ scale) for selecting variants into a polygenic score such that the area under the receiver operator characteristic curve (AUC) is maximized. A binary trait with prevalence 10% is assumed, with variants selected from a case/control study with equal number of cases and controls. Chip heritability of 40% (solid line) and 20% (dashed line) is distributed among 100,000 independent variants, of which 5% have normally distributed effects and the rest have no effect. The vertical line is at 50,000 cases and 50,000 controls, at which point over 95% of the maximum AUC is achieved.

As a final aspect of polygenic epidemiology discussed here, Mendelian randomization studies are increasingly using composite genetic scores to draw causal inferences from observational data [Nuesch et al., 2015; Thrift et al., 2015; Voight et al., 2012; Zhang et al., 2015]. The advantages are similar to those of individual prediction, in that a composite score can predict the intermediate trait to a greater accuracy than can single variants. A particular difficulty however is that Mendelian randomization assumes that the genetic instrument only affects the outcome through the exposure of interest. This assumption is increasingly likely to be violated as more variants enter the model [Burgess and Thompson, 2013; Palmer et al., 2012]. There is a stronger argument for restricting gene scores to robustly associated loci, but pleiotropy can still violate the assumption [Holmes et al., 2015]. In a highly polygenic score, variants with effects on the outcome but not on the exposure can create substantial bias [Evans et al., 2013]. Methods are now available to adjust Mendelian randomization analyses for known [Burgess and Thompson, 2015; Burgess et al., 2015] and unknown [Bowden et al., 2015] pleiotropic effects. Concerns about pleiotropy have so far discouraged the application of linear mixed models to Mendelian randomization, although in principle they could improve precision over polygenic scores.

The small effects of individual variants have caused the value of GWAS to be questioned [Manolio, 2013]. But bearing in mind that most complex traits are strongly heritable, the polygenic risk is highly informative taken *en masse* as a single risk factor. Confirmation is appearing through the applications of polygenic epidemiology discussed here, and further applications—such as identifying interactions, or mediation analysis—are sure to be developed in the near future. So far, the field has mainly developed from psychiatric genetics, in which progress in identifying and following up GWAS associations has been slower than in other areas. However, as seen here, applications are becoming common in cardiovascular, cancer, and immunological genetics. Other areas will add further insights: for example, population geneticists have used GWAS signals to demonstrate polygenic adaptation [Berg and Coop, 2014]. Linear mixed models are in principle the most powerful and accurate class of methods, with the principal challenges being the correct specification of the random effects distribution, and computational issues in large samples [Ge et al., 2015]. Polygenic risk scores, and LD scoring, offer simpler and faster approaches, requiring only summary statistics from completed GWAS, and generally incurring only a moderate loss of precision compared to linear mixed models. The variety of methods and applications now emerging in polygenic epidemiology will make this field a fertile ground for development in the coming years.
